# Association of prenatal medical risk with breastfeeding outcomes up to 12 months in the All Our Families community-based birth cohort

**DOI:** 10.1186/s13006-021-00413-0

**Published:** 2021-09-15

**Authors:** Natalie V. Scime, Amy Metcalfe, Alberto Nettel-Aguirre, Suzanne C. Tough, Kathleen H. Chaput

**Affiliations:** 1 Department of Community Health Sciences, Cumming School of Medicine, University of Calgary, Calgary, AB Canada; 2 Department of Obstetrics & Gynecology, Cumming School of Medicine, University of Calgary, Calgary, AB Canada; 3 Centre for Health and Social Analytics, National Institute for Applied Statistical Research, School of Mathematics and Statistics, University of Wollongong, Wollongong, NSW Australia; 4 Department of Pediatrics, Cumming School of Medicine, University of Calgary, Calgary, AB Canada

**Keywords:** Breast feeding, Pregnancy, high-risk, Pregnancy complications, Prospective studies, Logistic models, Survival analysis

## Abstract

**Background:**

Prenatal medical risk describes physical health issues or biological factors that predate or arise during pregnancy which heighten the risk of adverse outcomes, and often warrant specialized obstetric care. The influence of the nature and magnitude of prenatal risk on breastfeeding outcomes remains poorly understood. The objective of this study was to determine the association between prenatal medical risk and breastfeeding initiation and duration up to 1 year postpartum.

**Methods:**

We analysed a subset of data from the All Our Families longitudinal cohort (*n* = 2706) of women in Calgary, Canada who delivered a liveborn infant between 2008 and 2010. Data were collected from self-report questionnaires and medical records. Women with complete data on prenatal medical risk factors and breastfeeding outcomes were included in this analysis. Prenatal medical risk was operationalized as one integer score of risk severity and four binary risk types capturing pre-pregnancy characteristics, past obstetric problems, current obstetric problems, and substance use. Outcomes were breastfeeding initiation defined as the infant ever receiving breast milk, and duration operationalized as still breastfeeding at 4 months, at 12 months, and time to breastfeeding cessation in weeks. We used logistic regression and Cox regression with right censoring at 52 weeks or attrition to calculate odds ratios (OR) and hazard ratios (HR), respectively, adjusting for sociodemographic vulnerability, parity, mode of delivery, and gestational age.

**Results:**

Prenatal medical risk severity and type were not significantly associated with breastfeeding initiation, with the exception of pre-pregnancy risk type (OR 0.45; 95% CI 0.26, 0.77). Risk severity was associated with lower odds of breastfeeding to 4 months (OR 0.94; 95% CI 0.90, 0.99), 12 months (OR 0.93; 95% CI 0.87, 0.98), and earlier breastfeeding cessation (HR 1.05; 95% CI 1.02, 1.08). Associations with shorter breastfeeding length across the first postpartum year were observed for pre-pregnancy, current obstetric, and substance use risk types, but not past obstetric problems.

**Conclusion:**

Prenatal medical risk is associated with shortened duration of breastfeeding. Women with prenatal medical risk may benefit from the proactive arrangement of lactation support before and following delivery to promote continued breastfeeding.

**Supplementary Information:**

The online version contains supplementary material available at 10.1186/s13006-021-00413-0.

## Background

Breastfeeding is the optimal nutrition source for infants and has many established health benefits [1]. Breastfed infants have lower incidence of respiratory and digestive tract infections during childhood [2], as well as lower risk for asthma, type 1 or 2 diabetes, and obesity into adulthood compared to formula-fed infants [1]. Mothers who breastfeed their children experience lowered risk for breast and ovarian cancers and cardiovascular diseases [3, 4]. Breastfeeding is recommended as the primary source of nourishment until 6 months when complementary foods are introduced, and sustained for longer–up to 2 years and beyond–according to maternal preference [5, 6]. Identifying women who are at-risk for suboptimal breastfeeding is a salient clinical and public health goal.

Accumulating evidence suggests that women with prenatal medical risk are less likely to initiate and maintain breastfeeding than healthy women. Prenatal medical risk can be defined as one or more physical health issues or biological factors that predate or arise during pregnancy which heighten the risk of adverse perinatal outcomes, and often warrant specialized obstetric care. Risk factors shown to be associated with suboptimal breastfeeding, and particularly shorter breastfeeding duration, include gestational diabetes [7], hypertensive disorders of pregnancy [8], chronic disease such as inflammatory bowel disease and epilepsy [9–12], obesity [13], and cigarette use [14, 15]. For example, Kozhimannil et al. defined pregnancy complexity as taking blood pressure medication before pregnancy, having diabetes, or body mass index (BMI) greater than 30; among women intending to exclusively breastfeed, those with pregnancy complexities had 31% lower relative odds of exclusive breastfeeding at 1 week than those with healthy pregnancies [16]. Scime et al. found that maternal chronic diseases did not affect breastfeeding initiation or continuation to 6 months, but were associated with 2.5 times the odds of stopping exclusive breastfeeding before 6 months [12]. Using US surveillance data, Weiser et al. reported that light and moderate/heavy smokers during pregnancy had greater odds of failure to initiate breastfeeding (prevalence ratios 1.4 and 1.4, respectively) and ceased breastfeeding earlier (HRs 1.7 and 1.9, respectively) [15]. However, existing research only accounts for a small portion of criteria that would qualify a woman as having prenatal medical risk, including various maternal characteristics, pregnancy-related or chronic conditions, unhealthy lifestyle behaviors, and adverse reproductive history. Moreover, the influence of the nature and magnitude of prenatal risk on breastfeeding has been unexplored. Our objective was to determine the association between prenatal medical risk and breastfeeding outcomes from birth to 12 months.

## Methods

### Cohort selection

**Fig. 1:**
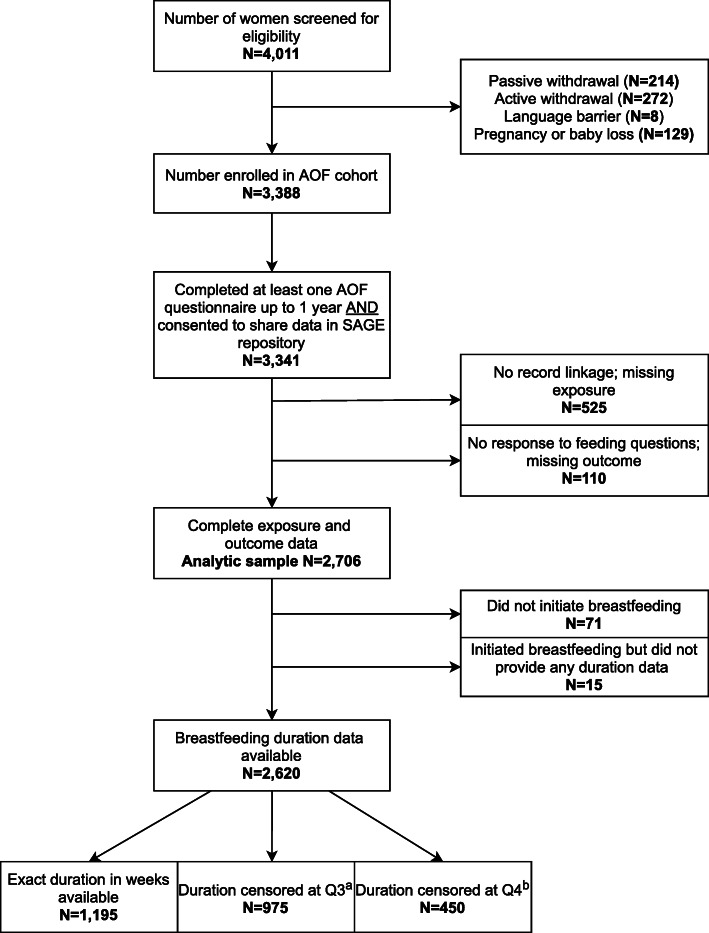
Flowchart of study sample. AOF = All Our Families study. SAGE = Secondary Access to Generate Evidence. Q3 = 4-month follow-up. Q4 = 12-months follow-up. aParticipants reported still breastfeeding at Q3 but did not provide duration data at Q4 (did not receive Q4). bParticipants reported still breastfeeding at Q4

We conducted a secondary analysis using a subset of data (*n* = 2706) from the All Our Families (AOF) longitudinal pregnancy cohort study in Calgary, Canada (*n* = 3388; see Fig. 1). Pregnant women < 25 weeks’ gestational age who could complete questionnaires in English, received prenatal care in Calgary, and were 18 years or older were recruited into the AOF study from a city-wide laboratory service, primary health care clinics, community posters, and by word of mouth between May 2008 and December 2010. Questionnaires wereadministered throughout the prenatal, postpartum, and childhood periods to collect information on family demographics, medical and obstetric history, lifestyle, psychosocial health, health behaviors (including infant feeding), health service use, and child health and development. The dataset for this analysis included maternal self-report data from the prenatal (< 25 weeks’ gestation and 34-36 weeks’ gestation) and postpartum (4 months, 12 months) follow-ups, as well as delivery hospitalization chart data deterministically linked to participants via personal health numbers [17]. Attrition in AOF is comparable to that of similar longitudinal cohort studies; participant response rates for the 4- and 12-month follow-ups were 90% (3057 responded / 3388 eligible) and 81% (1573 responded / 1942 eligible), respectively. The number of eligible participants at 12 months decreased due to the timing of questionnaire development and administrative delays in study implementation. Our analytic sample of 2706 consisted of AOF participants who delivered a liveborn infant and consented to medical chart linkage, excluding those with incomplete data for the study exposure or outcome. Ethics approval for the AOF study (REB #20821 and #22821) and for this secondary analysis (REB #18–0853) was received by the Conjoint Health Research Ethics Board at the University of Calgary.

### Exposure

Prenatal medical risk was measured using the Antepartum Risk Score (APRS), which is documented on participants’ delivery hospitalization records. The APRS is a clinical tool used to identify and manage the delivery of medically high-risk pregnancies in Alberta. This tool was introduced in 1986 and modified in 1992 to its current form with 46 risk factors that are weighted according to their relative importance for adverse outcomes (see Table 1) [19]. We derived variables for prenatal medical risk severity, category, and type from the APRS tool. One numerical score of prenatal risk severity was defined as the sum of the weights for each endorsed factor. Summary scores could theoretically range from 0 to 90 (if every item were endorsed); however, an upper limit of 22 has been reported previously [20]. One binary variable for prenatal risk category was defined as low medical risk for scores of 0–2 and high medical risk for a score of 3 or more [20]. Four binary prenatal medical risk types were each operationalized as presence of one or more factors pertaining to: pre-pregnancy characteristics defined as factors that existed before conception; past obstetric problems defined as a history of pregnancy or birth complications in previous pregnancies; current obstetric problems defined as the presence of pregnancy complications affecting the index pregnancy; and substance use defined as use of drugs, cigarettes, or alcohol in the index pregnancy. We excluded advanced maternal age from the list of pertinent factors for pre-pregnancy risk type despite its presence on the original APRS tool; all pre-pregnancy factors have a plausible detrimental association with both breastfeeding and perinatal outcomes except advanced age. Maternal age has a well-established detrimental association with perinatal outcomes [21], but positive association with breastfeeding [22], and thus exclusion of advanced maternal age in the pre-pregnancy risk type avoided biasing our result towards the null.

**Table 1:** The Antepartum Risk Score (APRS) Tool [18] used to measure prenatal medical risk

Score	Risk Factor	Score	Risk Factor
**Pre-pregnancy**		**Current Obstetrical**	
1	Age ≤ 17 at delivery^a^	2	Diagnosis of large for dates^c^
2	Age ≥ 35 at delivery	3	Diagnosis of small for dates^c^
1	Weight ≥ 91 kg^b^	2	Polyhydramnios or oligohydramnios
1	Weight ≤ 45 kg	3	Multiple pregnancy^a^
1	Height < 152 cm	3	Malpresentation (breech or transverse lie)
1	Diabetes – Controlled by diet only	2	Membranes ruptured before 37 weeks
3	Diabetes – Insulin used	1	Bleeding < 20 weeks
3	Diabetes – Retinopathy documented	3	Bleeding ≥ 20 weeks
1	Heart Disease – Asymptomatic (no affect on daily living)	2	Gestational hypertension
3	Heart Disease – Symptomatic (affects daily living)	1	Proteinuria ≥ 1+
2	Hypertension – 140/90 or greater	1	Gestational diabetes documented
3	Hypertension – Antihypertensive Drugs	3	Blood antibodies (Rh, Anti C, Anti K, etc.) ^c^
2	Chronic Renal Disease Documented	1	Anemia (Hgb< 100 gm. per L)
1	Other medical disorders e.g. epilepsy, severe asthma, lupus	1	Pregnancy > 41 weeks
3	Cervical surgery^c^	1	Poor weight gain (26-36 weeks <0.5kg/week or weight loss)
**Past Obstetric**		3	Major fetal anomaly^c^
3	Neonatal death(s)	3	Acute medical disorder (acute asthma, thyrotoxicosis, UTI, etc)^c^
3	Stillbirth(s)	**Substance Use**	
1	Abortion 12-20 weeks and birth weight <500 grams^d^	1	Smoker – anytime during pregnancy
1	Delivery at 20-37 weeks	3	Alcohol – ≥ 3 drinks on any one occasion during pregnancy
2	Cesarean section	3	Alcohol – ≥ 1 drink per day throughout pregnancy
1	Small for dates - 5th percentile	3	Drug dependent
1	Large for dates - 95th percentile		
1	RH Isoimmunization - unaffected infant^c^		
3	RH Isoimmunization - affected infant^c^		
1	Major fetal anomaly e.g. Chromosomal, Heart, CNS defect		

^a^ Factors not present in the AOF sample due to cohort exclusion criteria

^b^ Corresponds to a body mass index of ≥30 kg/m^2^ (obese) for women of average height

^c^ Due to data availability issues, these factors were included in the overall prenatal medical risk severity score and category variables but were not included in the risk type variables. Risk severity was extracted as a single number from the medical chart, whereas risk types were derived using binary variables for individual risk factors extracted from the chart

^d^ Includes spontaneous and therapeutic abortion

### Outcomes

Breastfeeding initiation was operationalized as a binary variable reflecting mothers’ responses to the question “Did you breastfeed or feed breast milk to your baby, even if only for a short time?” asked at 4 months postpartum. While data on breastfeeding in the first hour of life was also collected in AOF, high overlap (96%) between this variable and breastfeeding initiation underpinned our decision to solely report on breastfeeding initiation here. Breastfeeding duration was defined as mothers’ self-reported length of feeding their infant breast milk alone or in combination with other food sources [23]. At 4 months and 12 months, mothers were asked whether they were still breastfeeding, and only those that reported ceasing breastfeeding at either time point (*n* = 1195) were then asked to report their exact duration in weeks or months. Breastfeeding duration was operationalized using binary variables for breastfeeding at 4 months and 12 months, as well as a continuous variable for duration of breastfeeding in weeks up to 52 weeks. Duration was standardized into weeks using the conversion of 4.33 weeks / month [24].

### Covariates

Sociodemographic vulnerability (SDV) was operationalized using a composite binary variable [25], defined as one or more of: low education (high school or less), low household income (below $60,000 CAD [$59,000 USD in 2010], the eligibility threshold for subsidized housing at the time of recruitment), or being new to Canada (lived in Canada for less than 5 years). SDV intended to capture individuals more likely to experience barriers to healthcare access, engaging in healthy behaviors, or social supports, which may consequently impact prenatal health and breastfeeding [26, 27]. Parity was defined as primiparous or multiparous. Mode of delivery was defined as vaginal (spontaneous or instrumental) or Cesarean section (scheduled or unscheduled). Gestational age at birth was measured as number of completed weeks. Though often included as covariates in breastfeeding research, we excluded pre-pregnancy BMI because it was instead considered an exposure as part of the APRS tool; maternal age because of known multicollinearity with education and parity, which mediate > 75% of the effect of maternal age on breastfeeding outcomes [28]; and marital status because almost all (95%) AOF participants were married or co-habiting with their partner [17].

### Statistical analysis

Descriptive statistics were used to summarize the sample in comparison to the full AOF sample and recent population-based data on Alberta mothers. Multivariable binary logistic regression was used, as is appropriate for dichotomous outcomes, to model the association between prenatal medical risk and the odds of initiating breastfeeding and continuing breastfeeding to 4 months and 12 months yielding odds ratios (OR) and 95% confidence intervals (CIs). Three models were constructed to compare the impact of adjusting for a priori covariates on point estimates. The first model (M1) was a crude estimate containing only the prenatal risk status. The second model (M2) adjusted for demographic covariates, SDV and parity. The third model (M3) additionally adjusted for obstetric covariates, mode of delivery and gestational age. All covariates were binary with the exception of gestational age, which was modelled as a flexible continuous covariate [29].

Kaplan-Meier plots were used to visually compare the breastfeeding duration curves according to prenatal medical risk status. Multivariable Cox proportional hazards regression was used, as is appropriate for time to event outcomes, to model the association between prenatal risk and time to cessation of breastfeeding up to 52 weeks yielding hazard ratios (HR) and 95% CIs. Participants were right-censored at loss to follow-up or at 1 year if they were still breastfeeding. Given the decrease in participant eligibility for the 12-month follow-up, censoring at 4 months was common (*n* = 975; see Fig. 1); however, comparisons between the analytic sample and the 4-month censored subsample indicated no notable differences in sociodemographic or maternal characteristics (see Additional file 1). Three models with increasing covariate adjustment were constructed as outlined above. Additional details regarding our statistical approach are outlined in Additional file 2.

We calculated predicted probabilities (logistic regression) and median survival (Cox regression) from the M3 models for exposed and unexposed groups using a fixed demographically low-risk covariate profile – low SDV, primiparous, and vaginal delivery at 40-weeks’ gestation–to illustrate a conservative example of the differences in breastfeeding outcomes we observed in our models.

We conducted one sensitivity analysis to assess the impact of measurement error on our results. Breastfeeding duration was standardized into weeks using an alternative conversion of 4 weeks / month, to represent the intuitive approach that women might have used to convert between units when responding to questionnaires [24]. Data cleaning and analyses were completed using Stata v.15 in the SAGE virtual environment.

## Results

Sample characteristics in comparison to the full AOF cohort and recent Alberta statistics (2014–2018) on the maternal population are displayed in Table 2. The majority of women in our sample were between 25 and 34 years of age (70.9%), had some post-secondary education (90.2%), household income above $60,000 (84.2%), and were white (79.5%), and half were primiparous (49.5%). Approximately one quarter (26.3%) of infants were delivered via Cesarean section and 7.2% were born before 37 weeks’ gestation. Sample characteristics were fairly comparable to that of the full AOF cohort and Alberta maternal population in recent years; however, some vulnerable groups such as young mothers and those with lower education were under-represented.

**Table 2:** Comparison of characteristics among mothers in the analytic sample, AOF cohort, province of Alberta

Characteristic	Analytic sample %	Full AOF cohort (2008–2010) %	Alberta (2014–2018) %
Number of participants	2706	3388	NA
Maternal age			
24 or younger	6.2	9.0	15.0
25–34	70.9	71.4	65.8
35 or older	22.9	19.6	19.2
Maternal education			
High school or less	9.8	10.9	24.9
Some post-secondary	90.2	89.1	75.1
Household income			
Below $60,000	15.8	18.3	24.5
$60,000 or greater	84.2	81.7	75.5
White ethnicity	79.5	78.6	61.9
Lived in Canada < 5 years	9.4	–	9.8
Body mass index, Mean	24.4	24.3	24.6
Primiparous	49.5	49.0	41.2
Cesarean delivery	26.3	24.8	29.1
Preterm birth	7.2	7.0	6.8
Initiated breastfeeding	97.4	97.9	90.1
Breastfed to 4 months	80.4	–	71.8
Breastfed to 12 months	32.2	–	39.1

*AOF* All Our Families. *NA* Not applicable; values reported come from multiple data sources

Full cohort characteristics: [17, 30]

Alberta characteristics: [31, 32]

**Fig. 2:**
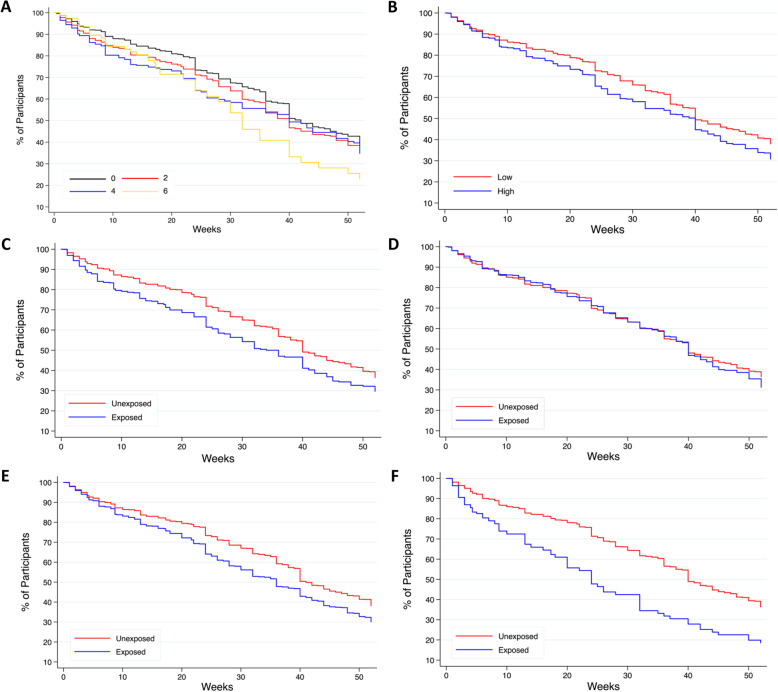
Kaplan-Meier plot of time to breastfeeding cessation. **A** prenatal medical risk severity (Crude hazard ratio [HR] 1.06; 95% CI 1.03, 1.09); **B** prenatal medical risk category (Crude HR 1.23; 95% CI 1.09, 1.39); **c** pre-pregnancy risk type (Crude HR 1.34; 95% CI 1.15, 1.55); **D** past obstetric risk type (Crude HR 1.07; 95% CI 0.93, 1.23); **E** current obstetric risk type (Crude HR 1.29; 95% CI 1.14, 1.45); **F** substance use (Crude HR 1.91; 95% CI 1.53, 2.38)

Prenatal medical risk was relatively common; the median prenatal risk severity score was 2 (range 0 – 16) and 34.1% of women were categorized as having high prenatal medical risk (score ≥ 3). The most common prenatal risk types were current obstetric (34.9%) and past obstetric (20.0%) problems. Breastfeeding duration outcomes according to prenatal medical risk status are shown in Fig. 2. Almost all women (97.4%) in the sample initiated breastfeeding following delivery, with 80.4% and 32.2% breastfeeding in any capacity to 4 months and 12 months, respectively, and a median breastfeeding duration of 35 weeks.

Results for breastfeeding initiation are outlined in Table 3. Prenatal medical risk severity, category, and type were not associated with breastfeeding initiation in fully adjusted models, with the exception of pre-pregnancy risk type associated with 0.45 lower relative odds of breastfeeding initiation (95% CI 0.26, 0.77). A significant crude association was observed for substance use (M1 OR 0.39; 95% CI 0.19, 0.80) risk type and lower odds of breastfeeding initiation, but this became non-significant after covariate adjustment (M2 OR 0.48; 95% CI 0.22, 1.05).

**Table 3:** Association between prenatal medical risk and breastfeeding initiation

	Breastfeeding initiation		
	M1 OR (95% CI)	M2 OR (95% CI)	M3 OR (95% CI)
Risk score	0.91 (0.83, 1.01)	0.95 (0.85, 1.05)	1.01 (0.90, 1.15)
Risk category			
High	0.79 (0.49, 1.28)	0.87 (0.54, 1.44)	1.17 (0.68, 2.01)
Risk type			
Pre-pregnancy	0.47 (0.28, 0.78)	0.43 (0.25, 0.73)	0.45 (0.26, 0.77)
Past obstetric	0.68 (0.40, 1.16)	1.01 (0.57, 1.78)	1.54 (0.78, 3.02)
Current obstetric	1.05 (0.64, 1.73)	0.94 (0.56, 1.60)	1.14 (0.64, 2.01)
Substance use	0.39 (0.19, 0.80)	0.48 (0.22, 1.05)	0.48 (0.22, 1.06)

M1 = model 1, crude. M2 = model 2, adjusted for sociodemographic vulnerability and parity. M3 = model 3, adjusted for sociodemographic vulnerability, parity, mode of delivery, and gestational age. All covariates were binary, except gestational age which was continuous

Risk scores represent each woman’s integer score on the Antepartum Risk Score tool (comprised of 46 weighted risk factors), and corresponds to the severity of prenatal medical risk

Hosmer and Lemeshow tests performed on all M3 logistic regression models were *P >* 0.05, indicating satisfactory model fit

Results for breastfeeding duration are outlined in Table 4. Prenatal medical risk severity was associated with shorter breastfeeding duration, and effect sizes were unchanged following full adjustment for covariates. The ORs were 0.94 (95% CI 0.90, 0.99) and 0.93 (95% CI 0.87, 0.98) per unit increase in severity score for breastfeeding to 4 months and 12 months, respectively, and the HR was 1.05 (95% CI 1.02, 1.08) per unit increase for earlier time to breastfeeding cessation. The significant associations between high prenatal medical risk and moderately lower odds of breastfeeding to 4 months and 12 months persisted after initial adjustment, but shifted towards the null and became non-significant after full adjustment (4 months, M3 OR 0.83, 95% CI 0.67; 1.04; 12 months: M3 OR 0.80; 95% CI 0.63, 1.02). High prenatal medical risk was also associated with earlier breastfeeding cessation after initial adjustment, and shifted towards the null but remained significant after full adjustment with an HR of 1.16 (95% CI 1.02, 1.33). Past obstetric risk type was not significantly associated with breastfeeding duration in crude or adjusted models. Pre-pregnancy and current obstetric risk types were associated with significant and moderate reductions in breastfeeding duration with consistent effect sizes at each stage of covariate adjustment. Pre-pregnancy risk type had an OR of 0.63 for breastfeeding to 4 months (95% CI 0.49, 0.80), 0.74 for breastfeeding to 12 months (95% CI 0.56, 0.99), and an HR of 1.31 for time to breastfeeding cessation (95% CI 1.13, 1.52) in fully adjusted models. Current obstetric risk type was associated with an OR of 0.74 for breastfeeding to 4 months (95% CI 0.59, 0.93), 0.67 for breastfeeding to 12 months (95% CI 0.52, 0.87), and an HR of 1.28 for time to breastfeeding cessation (95% CI 1.12, 1.46). Substance use risk type was significantly associated with the largest reduction in the odds of breastfeeding to 4 months (M3 0.47; 95% CI 0.32, 0.68), 12 months (M3 OR 0.36; 95% CI 0.20, 0.64), and in time to breastfeeding cessation (M3 HR 1.82; 95% CI 1.45, 2.29) in adjusted models.

**Table 4:** Association between prenatal medical risk and breastfeeding duration

	Breastfeeding to 4 months			Breastfeeding to 12 months			Time to Breastfeeding Cessation		
	M1 OR (95% CI)	M2 OR (95% CI)	M3 OR (95% CI)	M1 OR (95% CI)	M2 OR (95% CI)	M3 OR (95% CI)	M1 HR (95% CI)	M2 HR (95% CI)	M3 HR (95% CI)
Risk score	0.93 (0.89, 0.97)	0.92 (0.88, 0.96)	0.94 (0.90, 0.99)	0.92 (0.87, 0.96)	0.90 (0.86, 0.95)	0.93 (0.87, 0.98)	1.06 (1.03, 1.09)	1.06 (1.04, 1.09)	1.05 (1.02, 1.08)
Risk category									
High	0.76 (0.62, 0.92)	0.74 (0.60, 0.90)	0.83 (0.67, 1.04)	0.75 (0.60, 0.93)	0.72 (0.57, 0.90)	0.80 (0.63, 1.02)	1.23 (1.09, 1.39)	1.26 (1.11, 1.42)	1.16 (1.02, 1.33)
Risk type									
Pre-pregnancy	0.60 (0.47, 0.77)	0.60 (0.47, 0.77)	0.63 (0.49, 0.80)	0.70 (0.53, 0.93)	0.71 (0.53, 0.94)	0.74 (0.56, 0.99)	1.34 (1.15, 1.55)	1.33 (1.15, 1.54)	1.31 (1.13, 1.52)
Past obstetric	1.10 (0.86, 1.41)	0.97 (0.73, 1.27)	1.19 (0.88, 1.61)	0.90 (0.69, 1.16)	0.76 (0.57, 1.01)	0.87 (0.64, 1.18)	1.07 (0.93, 1.23)	1.17 (1.01, 1.37)	1.06 (0.89, 1.25)
Current obstetric	0.72 (0.59, 0.88)	0.74 (0.61, 0.91)	0.74 (0.59, 0.93)	0.67 (0.54, 0.84)	0.68 (0.54, 0.86)	0.67 (0.52, 0.87)	1.29 (1.14, 1.45)	1.27 (1.13, 1.44)	1.28 (1.12, 1.46)
Substance use	0.42 (0.29, 0.60)	0.47 (0.32, 0.68)	0.47 (0.32, 0.68)	0.36 (0.20, 0.63)	0.36 (0.20, 0.64)	0.36 (0.20, 0.64)	1.91 (1.53, 2.38)	1.83 (1.46, 2.30)	1.82 (1.45, 2.29)

M1 = model 1, crude. M2 = model 2, adjusted for sociodemographic vulnerability and parity. M3 = model 3, adjusted for sociodemographic vulnerability, parity, mode of delivery, and gestational age. All covariates were binary, except gestational age which was continuous

Risk scores represent each woman’s integer score on the Antepartum Risk Score tool (comprised of 46 weighted risk factors), and corresponds to the severity of prenatal medical risk

Time to breastfeeding cessation represents the amount of time, in weeks, between birth and self-report of stopping any breastfeeding (exclusively or in combination with other food sources)

Hosmer and Lemeshow tests performed on all M3 logistic regression models were *P >* 0.05, indicating satisfactory model fit

Predicted probabilities for breastfeeding to 4 months and 12 months and median breastfeeding duration are outlined in Additional file 3, translating ORs and HRs to illustrate the impact of prenatal medical risk on feeding outcomes for a demographically low-risk covariate profile. Sensitivity analysis using an alternative conversion of 4 weeks / month did not substantively differ from our original findings (results not shown).

## Discussion

Using prospective data from the AOF cohort, we have shown that prenatal medical risk is negatively associated with breastfeeding duration. Risk severity had an inverse relationship with duration, such that the odds of breastfeeding to 4 months and 12 months and overall duration decreased as severity scores increased. The type of prenatal medical risk was important; past obstetric risk type did not impact duration, whereas pre-pregnancy, current obstetric, and substance use risk types were associated with moderate to large reductions in breastfeeding duration. These associations persisted following covariate adjustment.

Our study compositely defined risk types and reinforces that exposed women have shortened breastfeeding duration across the first postpartum year, and reports novel information that this disparity increases in magnitude as a women’s risk severity increases. Using predicted probabilities for a demographically low-risk covariate profile, we illustrated that duration is approximately 1 month shorter among women with pre-pregnancy (HR 1.31) and current obstetric (HR 1.28) risk types, and approximately 3 months shorter among women with substance use (HR 1.82) risk type. For fixed time points, predicted probabilities suggested an absolute reduction of 7 – 13% in breastfeeding to 4 months (baseline prevalence of ~ 84%) and 7 – 20% in breastfeeding to 12 months (baseline prevalence of ~ 37%) across risk types. For breastfeeding initiation, we detected a significant inverse association with pre-pregnancy risk type and a non-significant reduction in the odds with substance use risk type; however, the absolute differences in predicted probabilities were small (1 – 2%). No studies could be located on adverse obstetric history and breastfeeding, possibly due to an assumption that any effect would be mediated by related characteristics of the index pregnancy. Correspondingly, we did not find significant associations between past obstetric risk type and breastfeeding outcomes.

In Canada, breastfeeding intention and initiation is high (90%) and in-hospital breastfeeding support is widely available [33], which may explain why we did not detect differences in initiation based on prenatal medical risk. In line with this thinking, we suspect that mixed evidence regarding breastfeeding initiation and prenatal medical risk may be confounded by geographic and cultural differences in hospital policies and feeding norms. Once women start breastfeeding, however, findings from our study and others suggest that prenatal medical risk presents barriers to continuing breastfeeding that are not explained by demographic characteristics or obstetric events. Prenatal medical risk may biologically disrupt lactation. Delayed onset of milk production (> 72 h after delivery) is more prevalent in women with diabetes [34], obesity [35], or prenatal alcohol use [36], and can have sustained negative effects on breastfeeding duration [37]. Systematic differences in milk components such as immune factors have been reported among women with preeclampsia [38] and those who smoke [39] compared to healthy controls, suggesting an interaction with mammary gland function. Future research should explore differences in lactational factors to elucidate the extent to which prenatal medical risk may be interfering with lactation potential. Women’s experiences likely also play a role. Qualitative reports from women with pregnancy complications or chronic illness emphasize protracted physical recovery from childbearing and concerns about medication safety during breastfeeding [40, 41]. Women with substance use disorders have discussed unpleasant withdrawal symptoms, stigmatizing interactions with health professionals, and complex mental health or social issues which may impede breastfeeding [42, 43]. Additional research on the experience of establishing and maintaining breastfeeding in women with prenatal medical risk is warranted to explore these interpretations further. Interestingly, we found that breastfeeding duration shortens as risk severity increases, implying that any biological or psychosocial underpinnings intensify as prenatal medical risk factors accumulate.

### Strengths and limitations

Our findings should be considered in light of several limitations. Because maternal mental health issues have been extensively studied in the context of breastfeeding [44], we focused exclusively on physical health issues here; however, this precluded us from commenting on the intersection between these types of conditions which represents an important avenue of future study. Feeding outcomes were self-reported and are subject to social desirability bias, and were not measured beyond 1 year in this cohort. Data on the frequency of feeds across all nutrition sources were not collected, and thus our definition for “breastfeeding” is heterogeneous and inclusive of token to exclusive breastfeeding. We used the APRS tool to inclusively measure prenatal medical risk; however, the criteria, weights, and validation of this tool have not been updated since tool revision in 1992. Nonetheless, in the absence of a generally agreed upon definition of prenatal medical risk or high-risk pregnancy, the APRS tool presented advantages in terms of comprehensiveness, availability, and contemporary usage in perinatal health research [45–47]. Participant attrition between 4 months and 12 months postpartum in this cohort was high, though the administrative explanation for this and our comparison between those censored at 4 months and the full sample indicate that attrition was likely random. Because of this attrition, we analyzed short-term breastfeeding at 4 months instead of 6 months in an effort to maximize sample size and use the fullest extent of breastfeeding data available. Residual confounding is possible because certain variables, such as marital status and maternal age, were excluded from analysis. Our analysis did not examine the role of modifiable factors such as medicine use, social support, pacifier use, and complementary feeding patterns [48, 49], which may differ by prenatal medical risk status and could thus explain or mediate some of the association we observed. Additional research on the influence of these factors in the context of high-risk pregnancy and breastfeeding would be beneficial for identifying intervention opportunities. Recruitment and pregnancy data collection for AOF participants took place over 10 years ago; however, maternal characteristics in AOF are fairly comparable to recent provincial statistics, supporting that data from this cohort is still relevant today. Finally, participation in the AOF cohort was voluntary, and generalizability to certain vulnerable demographic groups who are under-represented should be done with caution.

Strengths of the data source include prospective data collection and linkage to medical records, limiting the potential for recall bias or memory error. The AOF cohort is representative of the maternal population in Alberta and Canada, which supports the external validity of our findings [17]. We used several variables to operationalize prenatal medical risk and modelling techniques to confirm that results were robust to varying methodologic approaches.

## Conclusions

In summary, we have shown that prenatal medical risk is associated with shorter breastfeeding duration on average, even after accounting for sociodemographic background and obstetric events. Prenatal substance use in particular is associated with the largest reduction in breastfeeding duration among the risk types studied. Our findings lend to additional inquiry regarding the etiologic nature of this relationship, with a focus on pre-pregnancy, current obstetric, and substance use risk types, which would help pinpoint what specific interventions may be effective for rectifying the disparity we observed. As it stands, women with prenatal medical risk would likely benefit from additional support to optimize breastfeeding duration, particularly in the first 4 months before complementary foods are introduced. While the APRS tool is unique to Alberta, our work suggests that the presence of prenatal medical risk factors can cue health care providers to coordinate proactive feeding support. This may include additional counselling during pregnancy to establish breastfeeding goals, lactation support shortly following delivery, and prompt postpartum referral to public health or community-based supports. Additional knowledge on the reasons for this disparity will be important for ensuring supports are sensitive to the needs of women with prenatal medical risk.

## Supplementary Information

**Additional file 1: eTable 1.** Comparison of characteristics between the analytic sample and the subsample censored in our time to event analysis.

**Additional file 2: eTable 2.** Additional detail regarding our statistical approach.

**Additional file 3: eTable 3.** Predicted probabilities of breastfeeding outcomes and median breastfeeding duration from fully adjusted regression models.

## Data Availability

The datasets generated and / or analysed during the current study are available from SAGE, a secure data repository managed by the Alberta Centre for Child, Family and Community Research (https://policywise.com/resource/access-control-and-security/). Requests for data (All Our Families: S01–197845.4) are welcomed, and can be directed to data@policywise.com.

## Abbreviations

AOF: All Our Families
APRS: Antepartum Risk Score
BMI: Body mass index
CI: Confidence interval
HR: Hazard ratio
OR: Odds ratio
SAGE: Secondary Analysis to Generate Evidence
SDV: Sociodemographic vulnerability
