# Supplementary material for: Association of prenatal medical risk with breastfeeding outcomes up to 12 months in the All Our Families community-based birth cohort
Source: Int Breastfeed J. 2021 Sep 15;16:69. doi: 10.1186/s13006-021-00413-0 (PMC8442292; doi:10.1186/s13006-021-00413-0)
Supplement: Supplementary file 1 — Additional file 1: eTable 1. Comparison of characteristics between the analytic sample and the subsample censored in our time to event analysis. [file 13006_2021_413_MOESM1_ESM.docx]

**Additional File 1**

Association of prenatal medical risk with breastfeeding outcomes up to 12 months in the All Our Families community-based birth cohort (Scime et al.)

**eTable 1.** Comparison of demographics between the analytic sample and the subsample censored in our time to event analysis

| **Characteristic** | **Analytic sample**  **%** | **Q3 censored subsample**  **%** |
| --- | --- | --- |
| Number of participants | 2706 | 975 |
| Maternal age |  |  |
| 24 or younger | 6.2 | 6.1 |
| 25-34 | 70.9 | 72.7 |
| 35 or older | 22.9 | 21.2 |
| Maternal education |  |  |
| High school or less | 9.8 | 7.9 |
| Some post-secondary | 90.2 | 92.1 |
| Household income |  |  |
| Below $60,000 | 15.8 | 15.9 |
| $60,000 or greater | 84.2 | 84.1 |
| White ethnicity | 79.5 | 74.7 |
| Lived in Canada <5 years | 9.4 | 11.0 |
| Body mass index, Mean | 24.4 | 23.7 |
| Primiparous | 49.5 | 50.9 |
| Cesarean delivery | 26.3 | 24.4 |
| Preterm birth | 7.2 | 7.2 |
| APRS category |  |  |
| Low | 65.9 | 68.8 |
| High | 34.1 | 31.2 |
| APRS risk type |  |  |
| Pre-pregnancy | 16.7 | 14.0 |
| Past obstetrical | 20.0 | 19.0 |
| Current obstetrical | 34.9 | 32.6 |
| Substance use | 5.5 | 4.0 |

Q3=4 month postpartum questionnaire.
