# Supplementary material for: Association of prenatal medical risk with breastfeeding outcomes up to 12 months in the All Our Families community-based birth cohort
Source: Int Breastfeed J. 2021 Sep 15;16:69. doi: 10.1186/s13006-021-00413-0 (PMC8442292; doi:10.1186/s13006-021-00413-0)
Supplement: Supplementary file 2 — Additional file 2: eTable 2. Additional detail regarding our statistical approach. [file 13006_2021_413_MOESM2_ESM.docx]

**Additional File 2**

Association of prenatal medical risk with breastfeeding outcomes up to 12 months in the All Our Families community-based birth cohort (Scime et al.)

**eTable 2.** Additional detail regarding our statistical approach

| Covariates | All covariates were binary with the exception of gestational age, which was modelled using a restricted cubic spline with 4 knots at the 5^th^, 35^th^, 65^th^, and 95^th^ percentiles of the sample to allow for flexible and non-linear distribution. |
| --- | --- |
| Linearity | For all analyses, linearity of the effect of APRS scores on the log odds and log hazard of breastfeeding outcomes was assessed through visual inspection of lowess curves and tests for confirmation and departures of linear trend (based on modelling all values of the score as ordinal). |
| Censoring | Participants were right censored if they reported still breastfeeding at the 4-month follow-up but did not participate in the 12 month follow-up (censored at 17.3 weeks), still breastfeeding at the 12-month follow-up (censored at 52 weeks), or a duration of breastfeeding longer than 52 weeks on the 12-month follow-up (censored at 52 weeks). |
| Proportional Hazards Assumption | The proportional hazards assumption was assessed for the exposure and covariates using log-log plots of survival as well as smoothed HR plots and tests based on Schoenfield’s residuals. Proportional hazards were not violated by any variables except for SES, which was then incorporated into the models using stratification to allow for varying baseline hazards. |
