# Supplementary material for: Association of prenatal medical risk with breastfeeding outcomes up to 12 months in the All Our Families community-based birth cohort
Source: Int Breastfeed J. 2021 Sep 15;16:69. doi: 10.1186/s13006-021-00413-0 (PMC8442292; doi:10.1186/s13006-021-00413-0)
Supplement: Supplementary file 3 — Additional file 3: eTable 3. Predicted probabilities of breastfeeding outcomes and median breastfeeding duration from fully adjusted regression models. [file 13006_2021_413_MOESM3_ESM.docx]

**Additional File 3**

Association of prenatal medical risk with breastfeeding outcomes up to 12 months in the All Our Families community-based birth cohort (Scime et al.)

**eTable 3.** Predicted probabilities of breastfeeding outcomes and median breastfeeding duration from fully adjusted regression models

|  | Breastfeeding initiation, % | Breastfeeding to  4 months, % | Breastfeeding to  12 months, % | Median breastfeeding duration, weeks |
| --- | --- | --- | --- | --- |
| **Risk score** |  |  |  |  |
| 0 | 99.3 | 83.7 | 38.0 | 44 |
| 2 | 99.4 | 82.0 | 34.1 | 41 |
| 4 | 99.4 | 80.2 | 30.5 | 40 |
| 6 | 99.4 | 78.3 | 27.1 | 39 |
| **Risk category** |  |  |  |  |
| Low | 99.3 | 83.3 | 37.0 | 43 |
| High | 99.4 | 80.6 | 32.1 | 40 |
| **Risk type** |  |  |  |  |
| Pre-pregnancy |  |  |  |  |
| Unexposed | 99.5 | 84.1 | 37.4 | 44 |
| Exposed | 98.8 | 76.9 | 30.7 | 39 |
| Past obstetrical |  |  |  |  |
| Unexposed | 99.4 | 83.3 | 36.2 | 43 |
| Exposed | 99.6 | 85.6 | 33.0 | 41 |
| Current obstetrical | |  |  |  |
| Unexposed | 99.3 | 83.9 | 38.1 | 44 |
| Exposed | 99.4 | 79.4 | 29.3 | 39 |
| Substance use |  |  |  |  |
| Unexposed | 99.4 | 83.6 | 37.5 | 43 |
| Exposed | 98.7 | 70.4 | 17.7 | 31 |

Predicted values are derived from logistic regression and Cox regression models adjusted for adjusted for sociodemographic vulnerability, parity, mode of delivery, and gestational age.

Fixed demographically low-risk covariate profile used to calculate predicted values: low sociodemographic vulnerability, primiparous, vaginal delivery at 40 weeks gestation.
